# Intranasal Administration of KCNN2 Blocking Peptide Improves Deficits in Cognitive Flexibility in Mouse Model of Fetal Alcohol Spectrum Disorders

**DOI:** 10.1093/ijnp/pyaf055

**Published:** 2025-08-08

**Authors:** Shahid Mohammad, Li Wang, Masaaki Torii, Kazue Hashimoto-Torii

**Affiliations:** Center for Neuroscience Research, Children’s Research Institute, Children’s National Hospital, Washington, DC, United States; Center for Neuroscience Research, Children’s Research Institute, Children’s National Hospital, Washington, DC, United States; Center for Neuroscience Research, Children’s Research Institute, Children’s National Hospital, Washington, DC, United States; Department of Pediatrics, Pharmacology and Physiology, School of Medicine and Health Sciences, George Washington University, Washington, DC, United States; Center for Neuroscience Research, Children’s Research Institute, Children’s National Hospital, Washington, DC, United States; Department of Pediatrics, Pharmacology and Physiology, School of Medicine and Health Sciences, George Washington University, Washington, DC, United States

**Keywords:** fetal alcohol spectrum disorders, KCNN2, intranasal administration, prefrontal cortex, cognitive flexibility

## Abstract

**Background:**

Fetal alcohol spectrum disorders (FASD) show a myriad of cognitive and neurological deficits, with the prevalence estimated to be 1% to 5 % in children. To date, there are no effective treatments for these deficits in FASD. In a mouse model of FASD, daily intraperitoneal administration of a potassium calcium-activated channel subfamily N member 2 (KCNN2) blocking peptide has been shown to improve motor learning deficits due to upregulation of KCNN2 channels. This study investigates whether intranasal administration of a KCNN2 blocking peptide, Leiurotoxin-1 Dab7 (Lei-Dab7), can improve cognitive flexibility, specifically reversal learning deficits, in these mice.

**Methods:**

We utilized a mouse model of prenatal alcohol exposure. Cognitive flexibility was assessed using the water T-maze test at postnatal day 40. Lei-Dab7’s specificity and cytotoxicity were evaluated in vitro, and intranasal delivery efficiency was confirmed through immunohistochemistry, quantifying its distribution and binding to neurons with elevated KCNN2 expression in the prefrontal cortex.

**Results:**

Lei-Dab7 showed high specificity and negligible cytotoxicity in vitro. Intranasal administration efficiently delivered Lei-Dab7 to the prefrontal cortex, where it specifically bound to neurons expressing increased KCNN2 channels. Behavioral tests demonstrated that Lei-Dab7 significantly improved cognitive flexibility, reversing the deficits in the water T-maze test seen in ethanol-exposed mice, without apparent acute physiological adverse effects.

**Conclusions:**

Intranasal administration of KCNN2 blockers, such as Lei-Dab7, represents a promising, non-invasive therapeutic approach for treating cognitive inflexibility and possibly other cognitive dysfunctions associated with FASD.

Significance Statement Effective treatments for cognitive problems in neurodevelopmental disorders have been elusive. Studies using mouse models have shown that pharmacological blockade of KCNN2 channels may offer therapeutic effects for conditions such as fetal alcohol spectrum disorders (FASD) and Angelman syndrome. Despite this promise, the potent lead compounds have only been tested by systemic administration. Recent studies have found the benefit of intranasal drug administration for central nervous system disorders such as autism. We therefore tested this non-invasive approach with Lei-Dab7, a highly potent KCNN2 blocking peptide whose in vivo efficacy has not been investigated to our knowledge. Our results show that this peptide reaches the prefrontal cortex via intranasal administration and ameliorates cognitive inflexibility in a mouse model of FASD. These findings pave the way for the development of efficient and patient-friendly treatments for cognitive problems in FASD and other neurodevelopmental disorders.

## INTRODUCTION

Prenatal alcohol exposure (PAE) diversely affects the cognitive and behavioral development of offspring,^1–7^ causing conditions known as fetal alcohol spectrum disorders (FASD).^8–11^ According to the Centers for Disease Control and Prevention, about 10% of women in the United States consume alcohol while pregnant, suggesting that the estimated prevalence of FASD in 1% to 5% (weighted estimate of 3.1%-9.85%) of school-aged children is conservative.^12^ Despite its heavy impacts on public health and economy, no effective interventions are currently available for FASD.^12–16^

A mouse model of PAE at the peak of corticogenesis recapitulates several cognitive and behavioral abnormalities broadly observed in human patients with FASD,^17^^,^^18^ serving as a suitable model to address the mechanisms underlying these abnormalities and identify treatments for FASD targeting those mechanisms. Our previous studies have revealed that PAE causes increased expression of KCNN2 (potassium calcium-activated channel subfamily N member 2; also called KCa2.2 or SK2) channels in the postnatal mouse motor cortex,^17^^,^^19^ and *Kcnn2* knockdown in the motor cortex via RNA interference ameliorates the motor skill learning deficits in these mice with PAE.^17^

KCNN2 plays a key role in regulating neuronal excitability by controlling the medium afterhyperpolarization.^20^ Previous studies have shown that KCNN2 blockers have therapeutic effects to improve learning and memory in several mouse and rat models of neuropsychiatric disorders associated with increased KCNN2 expression.^21–24^ We have shown that intraperitoneal (i.p.) administration of tamapin, a small KCNN2-blocking peptide that crosses the blood brain barrier, mitigates the abnormal increase in the peak amplitude of medium afterhyperpolarization in the motor cortex of PAE mice and improves their motor learning deficits.^17^ Similar to tamapin, which is a peptide toxin isolated from the venom of the Indian red scorpion *Mesobuthus amulus*, Lei-Dab7 (Leiurotoxin-1 Dab7)^25^^,^^26^ is a mutated version of leiurotoxin, a peptide identified from the venom of the scorpion *Leiurus quinquestriatus hebraeus*. Lei-Dab7 blocks KCNN2 with higher affinity and selectivity than tamapin,^25^ making it an attractive therapeutic candidate. With high affinity, selectivity, and potency to target receptors and membrane proteins, many venom peptides and their mimetics are considered to have enormous potential as drugs or scaffolds for drug design.^27^

Intranasal administration is a promising approach for efficient delivery of peptides and proteins to the central nervous system for the treatment of neurological, memory, and cognitive disorders with minimal systemic effects.^28^^,^^29^ The non-invasive nature of this approach is particularly suited for treating pediatric patients. Although still limited, intranasal application of hormones such as oxytocin and insulin has been used successfully as an effective treatment for cognitive and neurological conditions.^30–33^ Given the expression and function of KCNN2 outside the central nervous system,^34^ intranasal application may serve as an ideal approach to deliver KCNN2 blocking peptides for the treatment of cognitive problems in FASD.

In this study, we first report cognitive inflexibility in mice with PAE and that the phenotype is associated with elevated expression of KCNN2 in a part of the prefrontal cortex (PFC). We next demonstrate by in vitro assays that Lei-Dab7 inhibits KCNN2 channels highly specifically, with no detectable cytotoxicity. Furthermore, we show that intranasally administered Lei-Dab7 reaches the PFC and improves cognitive inflexibility in mice with PAE.

## METHODS

### Animals

All mice (CD-1) used in this study were purchased from Charles River Laboratories and were maintained in-house on a light–dark cycle (lights on 6:00 AM to 6:00 PM) at a constant temperature (22 ± 1°C). Pregnant mice received i.p. injections of 25% ethanol in PBS at 4.0 g/kg/day or PBS only as the control at embryonic day (E) 16 and E17. The dam’s blood alcohol concentration ranges from approximately 140 to 300 mg/dL (from 30 minutes after injection at E16 to 1 hour after injection at E17).^35^ All procedures were approved by the institutional animal care and use committee of the Children’s National Hospital.

### Immunohistochemistry

Mice were deeply anesthetized with isoflurane (Henry Schein, OH) and perfused through the ascending aorta with ice cold 4% paraformaldehyde (PFA) following a standard laboratory protocol. Brains were collected and post-fixed in 4% PFA at 4°C overnight, followed by incubation in 10% and 30% sucrose in PBS at 4°C for 24 hours. Coronal sections at 60 μm thickness were made using a cryostat (CM3050S; Leica, Germany). Free floating sections were incubated with hydrogen peroxide in methanol (1:4) solution at −20°C for 20 minutes to inactivate endogenous peroxidase activity. After washing with PBS containing 0.2% Tween 20 3 times, sections were incubated with blocking buffer (2% bovine serum albumin in PBS containing 0.2% Tween 20) for 30 minutes at room temperature. Sections were then incubated with the primary antibody [rabbit anti-KCNN2 (1:500) (Abcam, MA), mouse anti-NeuN (1:500) (Abcam, MA), goat ant-GFAP (1:500) (Abcam, MA), goat anti-IBA1 (1:500) (Abcam, MA)] diluted in the blocking buffer overnight at 4°C. Incubation with the secondary antibody [HRP-conjugated IgG (1:500), or biotin-conjugated IgG (1:200) (Jackson ImmunoResearch, PA)] lasted 3 hours at room temperature. ABC (Thermo Fisher Scientific, NY) or TSA kit (PerkinElmer, MA) was used for signal amplification. 4’,6-diamidino-2-phenylindole, dihydrochloride (DAPI) (1:10000) (Sigma Aldrich, MO) was used for nuclear counterstaining. Labeled sections were imaged using an Olympus confocal microscope equipped with a digital camera. Brightness of the images was adjusted using Image J and Photoshop (Adobe Systems, CA).

### Water T-maze Test

Tests were conducted in a Plexiglas T-maze filled with warm tap water (24 ± 1°C) at 6 cm depth. A clear platform (7.5 cm × 7.5 cm) was placed under the water surface at the end of the T-maze’s left or right arm. Mice were first exposed to the T-maze without the platform, then released from the start arm and allowed to explore for 30 seconds. This pretest procedure was repeated 6 times with 15 minutes inter-trial intervals to identify turning bias. Next, for testing of spatial learning, the platform was placed at the end of the goal arm on the opposite side from the animal’s preferred side. Mice were released from the start arm, and the trial was ended when the animal climbed onto the platform for 2 seconds or after 60 seconds elapsed. The response was counted as correct if the mouse reached the platform without entering the incorrect arm. Reaching the wrong arm was counted as an error response. Dependent measures include the latency to reach the platform and the days to reach the criterion. The criterion for acquisition of procedural/spatial learning was 7 or 8 correct responses per session. The reversal learning test was started from a day after the successful acquisition of the spatial learning task for each mouse. The same procedure as for the spatial learning test was used except that the platform was placed at the end of the opposite arm from that used for the spatial learning test. The same dependent measures were used in the analysis of reversal learning as in the analysis of spatial learning.

### Forced Swim Test

To test behavioral despair or depressive-like behavior, we conducted the forced swim test following a previous protocol.^36^ The mouse was placed in an inescapable transparent glass beaker (25 cm tall and 12 cm diameter) filled with warm tap water (24 ± 1°C) to a depth of approximately 20 cm. The mouse’s behavior was recorded by a digital video camera placed at a distance of approximately 40 cm from the glass beaker edge. The last 4 minutes of the 6-minute test period were used for analysis. Mobility (swimming) and immobility durations were measured as the time the mouse was freely swimming and the time the mouse was floating or gently padding with 1 foot (struggling), respectively.

### Thallium Flux Assay

The effect of tamapin and Lei-Dab7 to block the KCNN2 channel was assessed by the thallium flux (potassium ion surrogate) assay using FLIPR Potassium Assay KIT (Molecular Devices, CA) and a FLIPR TETRA fluorescence imaging plate reader (Molecular Devices, CA). Human embryonic kidney (HEK) 293 cell line stably expressing human KCNN2 (Charles River, MA) were plated on BioCoat Poly-D-Lysine 384-well Black/Clear Plates (BD Biosciences, NJ) at 15 000 to 30 000 cells per well and incubated at 37°C overnight or until cells form near confluent monolayer. A baseline fluorescence was recorded during the pre-incubation period, followed by activation in the stimulus buffer (K^+^-free buffer with 5 mM Tl^+^). The effects of tamapin and Lei-Dab7 on KCNN2 to inhibit KCNN2 channel were evaluated at 4 different concentrations (n = 4) each, based on fluorescence changes due to the treatments. The signal elicited by the respective positive control agonist [1 μM A23187 (Tocris, UK)] was set to 0% inhibition, and the signal in the presence of the respective positive control antagonists [3 μM UCL1684 (Tocris, UK)] was set to 100% inhibition. The IC_50_ values were defined as the concentration at which the percent inhibition equals 50.

### Cell Viability Assay

Potential cytotoxicity of Lei-Dab7 was assessed by an ATP detection assay. Jurkat (clone E6-1) cells were used as they express KCNN2 channels constitutively and have been used to assess the cytotoxicity of compounds that block KCNN2.^37^^,^^38^ After recovery from cryopreservation, cells were plated into V-bottom, 96-well plates (2 × 10^4^ cells per well), and the compound (Lei-Dab7) or control (negative control: media alone or 10% PBS in media; positive control: approximately 1% sodium dodecyl sulfate in media) was added into the culture media. After incubation for 24 hours, cells were assessed for viability using a CellTiter-Glo 2.0 kit (Promega, WI). Briefly, CellTiter-Glo 2.0 reagent was thawed and warmed to approximately 22°C, then mixed by gentle inversion. Once thoroughly mixed, 100 μL of reagent was distributed into each well, and the plates were gently mixed for approximately 2 minutes on an orbital shaker for cell lysis. The plates were then incubated for approximately10 minutes at room temperature to stabilize the luminescent signal. The amount of light produced from each well was quantified using a Cytation 5 plate reader (BioTek, VT). Cell viability was calculated as a percentage of the mean of the negative control tissues for each well.

### Intranasal Administration of Lei-Dab7

Lei-Dab7 or biotinylated Lei-Dab7 (Smartox Biotechnology, France) was dissolved in PBS and administered to mice intranasally at 3.0 μg/kg/day. Vehicle (PBS) only was administered as the control. Briefly, the mouse was held softly on the neck to avoid any movement during drug administration. A 20-μL pipette was used to deliver 3 to 5 μL drug at a time. The tip was placed close enough to the mouse’s nostril at a 45-degree angle, and the drug was slowly ejected into the nostril. The mouse was let to inhale for 3 to 5 seconds and then left in a cage for 5 minutes to fully inhale the drug. This process was repeated until the required amount of drug was administered in a few minutes. The alternate nostril was used to deliver the drug each time to increase the efficiency of administration. Successful delivery by this method was confirmed by detecting biotinylated Lei-Dab7 in the brain, which was collected and immersion-fixed with 4% PFA at 15 minutes after intranasal administration using ABC (Thermo Fisher Scientific, NY) and TSA (PerkinElmer, MA) kits. For the analysis on the effect of treatment, Lei-Dab7 was administered daily throughout the reversal learning test phase of the water T-maze test. Physiological monitoring was recorded at 30 minutes and 24 hours post administration by using PhysioSuite (Kent Scientific, CT).

### Quantification of Immunolabeled Cells

KCNN2, NeuN, GFAP, IBA1, and biotin-positive cells were quantified. All images were taken by confocal microscopy (Olympus), keeping exposure settings constant. Allen Brain Atlas was used to define different brain regions visualized by DAPI staining, and single-plane images from defined regions were used for the quantification. The researcher conducting the quantification was blinded to treatment groups throughout the analysis to minimize bias. The size of the region of interest was determined for each analysis to include a sufficient number of labeled cells with clear and detailed staining. An initial threshold was applied using the built-in thresholding plug-in in ImageJ, and the threshold was then manually adjusted by visually inspecting the intensity of the background and stained areas of the image, considering the expected cell size and shape. The presence of DAPI-labeled nuclei was also confirmed. The same threshold was applied across all images for the same protein of interest whenever appropriate. The immunolabeled cells were then counted within a region of interest using the cell counter plugin in ImageJ. To ensure consistency and reproducibility, quantification was performed across multiple sections and biological replicates.

### Data Analysis

Equal or nearly equal numbers of female and male mice from at least 3 litters were included in each analysis unless otherwise stated in the figure legend [the sample size for each sex (5 or less), however, was too small to provide sufficient power to determine significance for each sex].

All data compared were collected at the same time. For the data that passed the D’Agostino & Pearson normality test, we performed 2-tailed Student *t*-test, 1-way ANOVA, or 2-way ANOVA followed by Šidák test as described at each analysis using Graphpad Prism version 10.0 (GraphPad Software, CA). In the cases we observed a statistically significant interaction between independent variables in 2-way ANOVA, we reported simple main effects using Graphpad Prism version 10.0 (GraphPad Software, CA). *P* < .05 was considered statistically significant.

## RESULTS

### PAE Causes Deficits in Cognitive Flexibility in Mice

**Figure 1:**
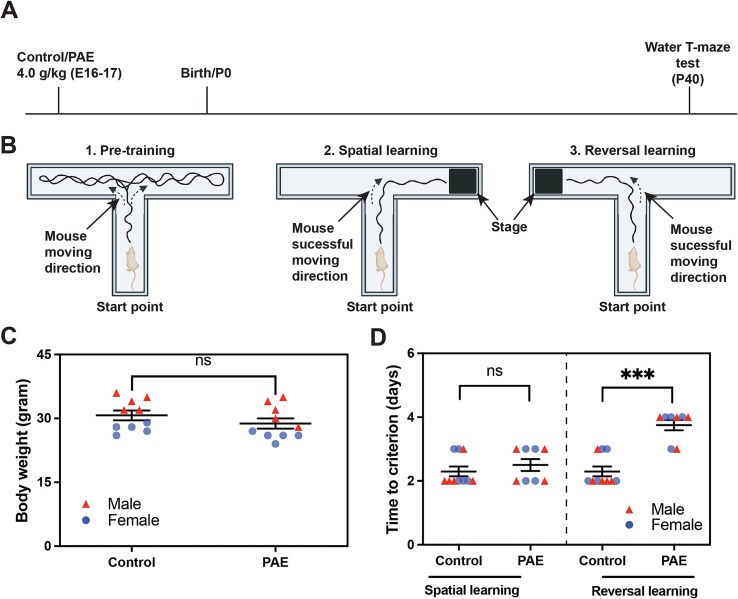
Prenatal alcohol exposure (PAE) causes deficits in reversal learning in mice. (**A**) Experimental timeline. (**B**) Scheme of water T-maze test. (**C**) Body weight is unaffected by PAE; *P* = .14 by 2-tailed Student *t*-test (n = 10 animals from 5 litters per group [including both males and females shown in red triangles and blue dots, respectively]). (**D**) T-maze test shows normal spatial learning but impaired reversal learning in PAE mice. *P* = .42 (ns: not significant) (spatial learning), and ^***^P < .001 (reversal learning) by 2-tailed Student *t*-test (control: n = 10 animals from 5 litters, PAE: n = 8 animals from 4 litters [including both sexes]). Data are presented as the mean with standard error.

Previous studies have reported that patients with FASD show object and spatial memory deficits^39–41^ as well as cognitive inflexibility.^40^^,^^42^ To address the mechanism underlying these abnormalities, we examined the impact of prenatal exposure to alcohol on spatial learning and reversal learning, which requires cognitive flexibility, disengaging from ongoing behavior, and adapting new environment/stimulus, in mice using the water T-maze test. Mice were exposed to ethanol (at 4.0 g/kg body weight; PAE mice) or PBS (control mice) in utero at embryonic (E) days 16 and 17, during which neurons in upper layers of the cerebral cortex are predominantly generated,^43^ and the water T-maze test was performed at postnatal (P) day 40 (Figure 1A, B).

Before the water T-maze test, we performed the forced swim test, which confirmed that these mice had no depressive-like behaviors that could confound the results of water T-maze test; we observed no significant differences in swimming and immobility times between control and PAE mice (Figure S1). Body weight did not differ between the control and PAE mice (Figure 1C). In the water T-maze test, we found no differences in spatial learning between the control and PAE mice. However, PAE mice showed deficits in reversal learning compared with the controls; PAE mice required more time (days) to learn the location of the stage after switching sides in the water T-maze (Figure 1D).

### KCNN2 Is Upregulated in Layer V of the PFC in PAE Mice

KCNN2 is known to play important roles in learning and memory.^44^^,^^45^ Our previous study has revealed significant KCNN2 upregulation in the primary motor cortex in PAE mice observed at P30,^46^ and the KCNN2 expression level is negatively correlated with the motor learning ability of these mice.^46^ Notably, we also found that the deficits in motor skill learning can be mitigated by postnatal i.p. administration of a KCNN2 blocker to PAE mice.^46^ However, whether altered KCNN2 expression is involved in the deficit in cognitive flexibility in PAE animals was unknown.

**Figure 2:**
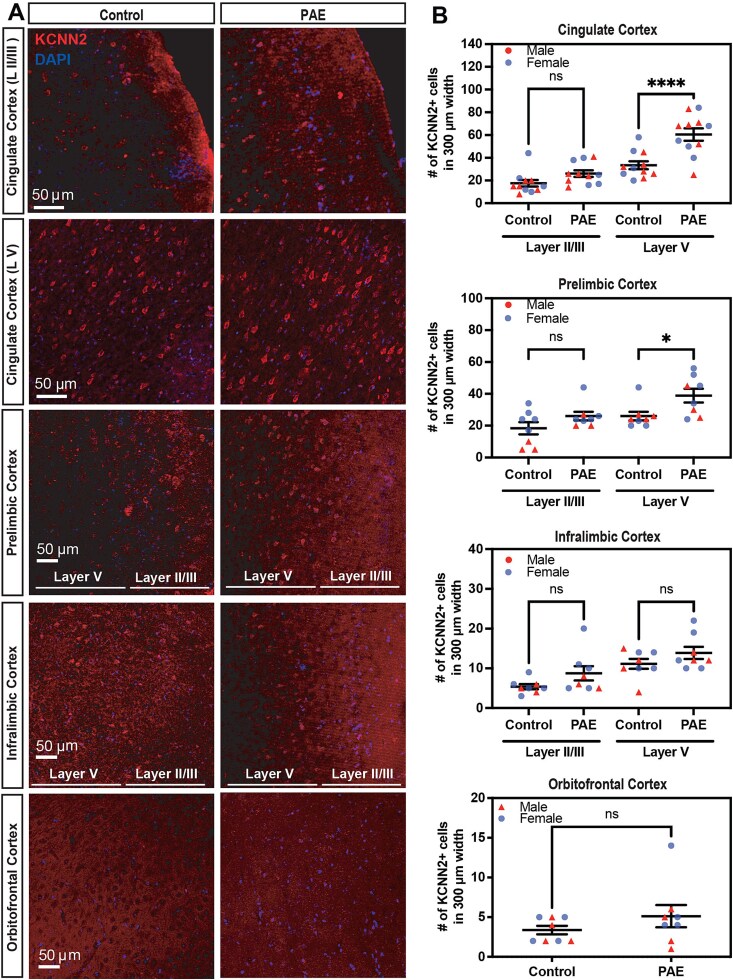
Increased expression of potassium calcium-activated channel subfamily N member 2 (KCNN2) in the prefrontal cortex (PFC) of PAE mice. (**A**) Immunohistochemistry reveals that the number of KCNN2 expressing cells is higher in layer V of the indicated cortical regions in PAE mice than in control mice at P40. In the orbitofrontal cortex, layer II/II and V were not analyzed separately due to its less clear laminar structure. (**B**) Quantification of KCNN2^+^ cells in layer II/III and V in the indicated cortical regions in control and PAE mice. A significant interaction between the effect of prenatal exposure and cortical layers was found by 2-way ANOVA [F(1,40) = 5.77, *P* = .02, n = 11 animals from 6 litters per group [including both sexes], in the cingulate cortex, where significant increase of KCNN2^+^ cells in PAE mice is observed in layer V (^****^P < .0001 by simple effect test). Significant main effects of exposure and cortical layers were found by 2-way ANOVA in the prelimbic cortex [F(1,28) = 8.67, *P* = .006 and F(1,28) = 8.67, *P* = .006, respectively] and infralimbic cortex [F(1,28) = 4.94, *P* = .03 and F(1,28) = 15.58, *P* = .005, respectively] (n = 8 animals from 4 litters per group [including both sexes]). Significant increase of KCNN2^+^ cells in PAE mice is observed in layer V in the prelimbic cortex (^*^P = .03 by Šidák test). Significant increase of KCNN2^+^ cells in PAE mice is also observed in the orbitofrontal cortex (^*^P < .05 by 2-tailed Student *t*-test) (n = 8 animals from 4 litters per group [including both sexes]). All data are presented as the mean with standard error.

We therefore performed immunohistochemistry for KCNN2 and quantified its expression in the PFC, the brain region essential for reversal learning,^47^ at P40. Our results revealed a significant increase in the number of KCNN2^+^ cells in layer V of both cingulate and prelimbic areas in PAE mice compared with control mice (Figure 2A, B). Similarly, the orbitofrontal and infralimbic areas showed a trend toward an increase in the number of KCNN2^+^ cells, although this did not reach statistical significance (Figure 2A, B). The upper layers also exhibited a similar trend (Figure 2A, B). Double staining for KCNN2 and a cell type–specific molecular marker—NeuN for neurons, GFAP for astrocytes, and IBA1 for microglia—revealed that the increase in KCNN2 expression in the PFC of PAE mice was primarily in neurons (Figure S2).

Previously, we showed that there was no significant change in KCNN2 expression in the hippocampus, a key brain region that mediates spatial learning, in PAE mice at P30.^46^ This aligns with the evidence that spatial learning, which primarily depends on hippocampal function, remains intact in PAE (Figure 1). The dorsal subregion of the striatum, which receives major input from the PFC and is involved in both reversal learning and spatial learning,^4^^,^^48^^,^^49^ also did not show significant changes in KCNN2 expression in PAE mice, at least at P30.^46^

### KCNN2 Blocker, Lei-Dab7, Is Efficiently Delivered to the PFC via Intranasal Administration

The increased expression of KCNN2 in the PFC of PAE mice suggested that KCNN2 blockade might mitigate the deficits in cognitive flexibility in PAE mice. In our previous study, we showed that i.p. administration of a KCNN2 channel blocker, tamapin, mitigated the deficits in motor skill learning in PAE mice.^46^ In the current study, we used another KCNN2 blocker, Lei-Dab7 (a synthetic derivative of leiurotoxin), a peptide isolated from the venom of the yellow scorpion (*L. quinquestriatus hebraeus*). Lei-Dab7 exhibits higher selectivity for KCNN2 than tamapin.^50^ We evaluated intranasal administration, as a non-invasive approach with more clinical relevance than i.p. injection, for the delivery of Lei-Dab7 to the brain.

To determine the dose of Lei-Dab7 to be tested, we compared the effect of Lei-Dab7 and tamapin in blocking human KCNN2 expressed in HEK293 cells using a thallium-based potassium ion channel assay. The IC_50_ value of Lei-Dab7 on KCNN2 (IC_50_: 0.72 μM) was >10 times higher than that of tamapin (IC_50_: 0.05 μM) (Figure S3). Lei-Dab7 did not show any cytotoxicity in 24-hour culture of Jurkat (clone E6-1) cells even as high as at 100 μM assessed by the ATP cell viability assay. Based on the effective dose of tamapin in mitigating motor skill–learning deficits in PAE mice (15 μg/kg body weight via i.p.)^17^ and higher brain uptake of therapeutics by intranasal delivery than by i.p. delivery, we chose a dose of 3.0 μg/kg body weight to test the effect of intranasal administration of Lei-Dab7.

**Figure 3:**
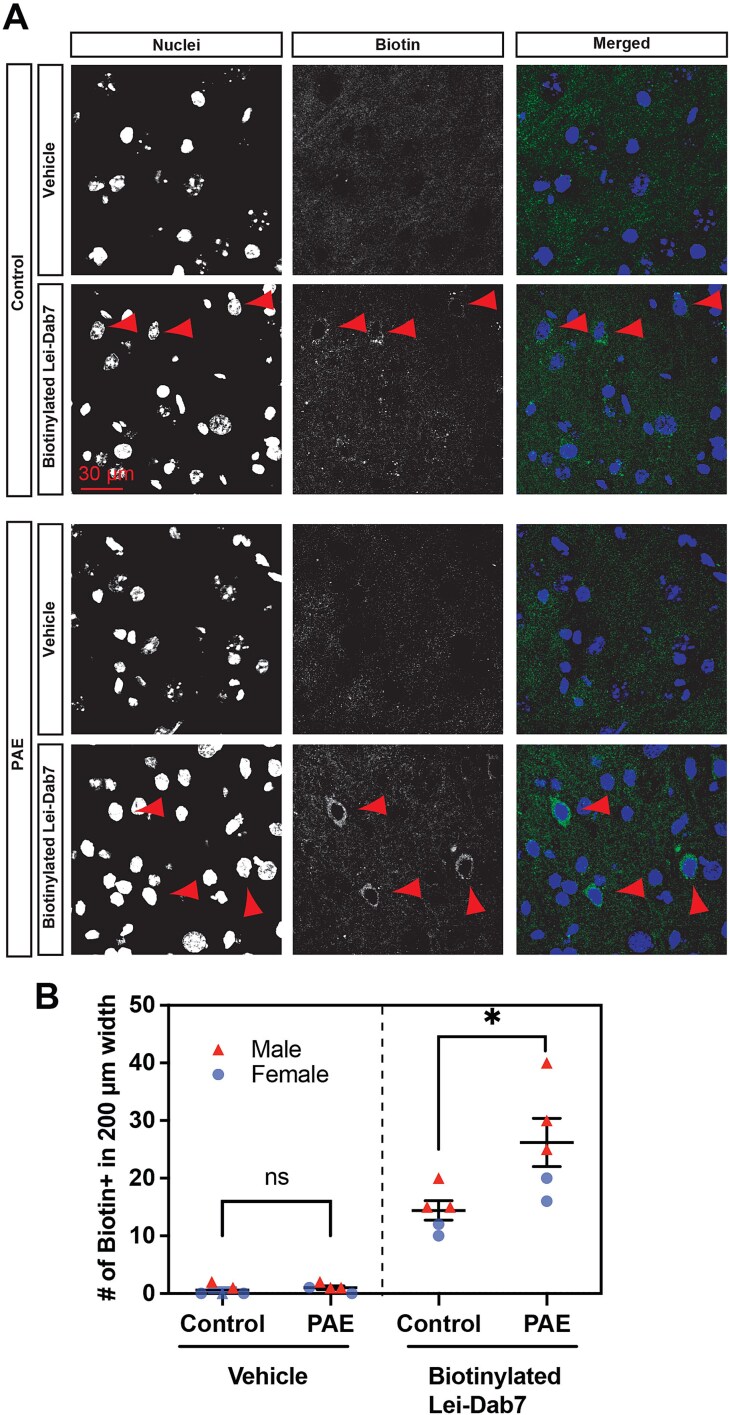
Intranasally administered Leiurotoxin-1 Dab7 (Lei-Dab7) reaches the PFC. Biotinylated Lei-Dab7 (or vehicle-only control) was administered intranasally to control and PAE mice at P40. Biotinylated Lei-Dab7 was detected 15 minutes post administration by immunohistochemistry for biotin (green). Cell nuclei were labeled by 4’,6-diamidino-2-phenylindole, dihydrochloride (DAPI) (blue). (**A**) Biotinylated Lei-Dab7 is detected strongly in a subset of neurons (arrowheads) in layer V of the PFC (the cingulate area is shown), especially in PAE mice. (**B**) The number of neurons with biotinylated Lei-Dab7 binding in their soma is increased in layer V of the PFC in PAE mice compared with control mice. ^*^P = .03 by 2-tailed Student *t*-test (n = 5 animals from 3 litters per group [including both sexes]). Data are presented as the mean with standard error.

**Figure 4:**
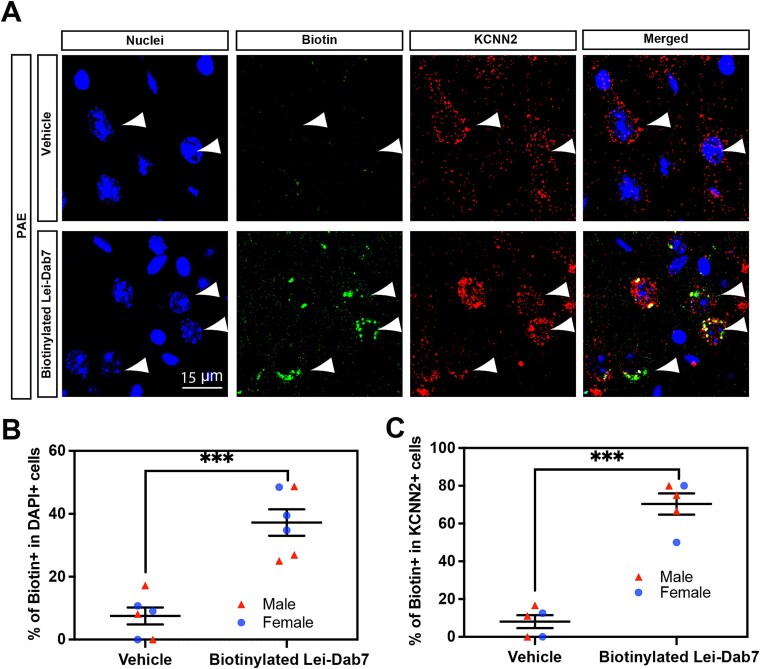
Lei-Dab7 binds to KCNN2 in PAE mice. Biotinylated Lei-Dab7 or the vehicle was administered intranasally to PAE mice at P40. The PFC tissue was fixed 15 minutes later. (**A**) Immunohistochemistry shows that biotinylated Lei-Dab7 (green) colocalizes with KCNN2 protein (red) in layer V cells in the PFC (arrowheads) (the cingulate area is shown). Cell nuclei were labeled by DAPI (blue). (**B**) Approximately 37% of all cells are labeled with biotinylated Lei-Dab7 in layer V in the PFC in PAE mice. ^***^P = .0001 by 2-tailed Student *t*-test (n = 6 animals from 3 litters per group [including both sexes]). (**C**) Most of the KCNN2^+^ cells are co-labeled with biotinylated Lei-Dab7 in layer V of the PFC in PAE mice. ^***^P < .001 by 2-tailed Student *t*-test (n = 5 animals from 3 litters per group [including both sexes]). Data are presented as the mean with standard error.

To determine whether the administered Lei-Dab7 reaches the PFC, biotinylated Lei-Dab7 (3.0 μg/kg body weight; with biotin conjugated to its N terminus) or vehicle (PBS) as a control was intranasally administered at P40. Fifteen minutes after administration, brains were fixed, and Lei-Dab7 binding was examined in brain slices using streptavidin-mediated biotin detection method. Biotinylated Lei-Dab7 was detected in layer V of the PFC, with a larger number of labeled cells in PAE mice than in control mice (Figure 3A, B). Among the labeled cells, Lei-Dab7 binding was well co-localized with KCNN2 expression (Figure 4A), consistent with the increased KCNN2 expression in the PFC in PAE mice (Figure 2). These results demonstrate that intranasally delivered Lei-Dab7 efficiently reaches the target in the brain.

No severe toxicity of intranasally delivered Lei-Dab7 was observed in our assessment on 3 primary physiological measures—pulse oximetry, heart rate, and body temperature—at either 30 minutes or 24 hours post administration (Figure S4).

### KCNN2 Blockade Improves Reversal Learning Deficits in PAE Mice

**Figure 5:**
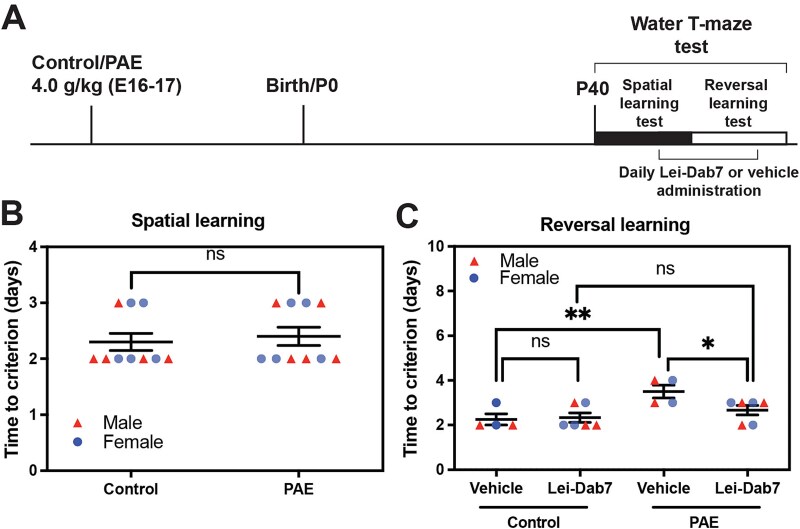
Lei-Dab7 improves deficits in reversal learning in PAE mice at P40. (**A**) Experimental timeline. After the spatial learning test, mice were divided into 2 groups. One group received Lei-Dab7 (3.0 μg/kg body weight/day), and the other group received vehicle only (control treatment). Both treatments were administered intranasally once daily, starting 24 hours before the first reversal learning trial and continuing until the day before the final test was completed. Siblings from the same mother were used as the control for the Lei-Dab7 treatment. (**B**) In these PAE mice, spatial learning is not affected (n = 10 animals from 5 litters per group [including both sexes]). (**C**) The reversal learning deficit in these PAE mice is improved by Lei-Dab7 administration (see PAE-vehicle vs PAE-Lei-Dab7). A significant effect of exposure [F(1,16) = 10.90. *P* = .005] but not of treatment [F(1,16) = 2.45, *P* = .14] was found by 2-way ANOVA. ^*^P = .03 and ^**^P = .004 by Šidák test (vehicle: n = 4 animals from 3 litters, Lei-Dab7: n = 6 animals from 3 litters, for both control and PAE groups [including both sexes]). Data are presented as the mean with standard error.

To test whether intranasal Lei-Dab7 administration improves the reversal learning deficit in mice with PAE, water T-maze test was performed. Spatial learning was first assessed starting from P40; after the spatial learning test phase, the mice were divided into 2 groups: group 1 received Lei-Dab7 (3.0 μg/kg body weight/day) and the other received vehicle only (control) intranasally, daily throughout the reversal learning test phase (Figure 5A). Again, no differences between control and PAE mice were observed in their spatial learning ability (Figure 5B). In PAE mice, impaired reversal learning was significantly improved to the level comparable with that in control mice by Lei-Dab7 administration (Figure 5C). Lei-Dab7 administration showed no effects on reversal learning in control mice (Figure 5C).

## DISCUSSION

In this study, we found the impact of PAE on cognitive flexibility caused deficits in reversal learning. This aligns with previous findings observed in FASD patients and other PAE animal models.^40^^,^^42^^,^^51^ Depressive-like behaviors as measured by the forced swim test were not affected in our PAE model (Figure S1). This is in contrast to a previous study that reported depressive-like behaviors, including increased immobility in the forced swim test, in a mouse model of chronic PAE.^52^ This difference may be due to the difference in the exposure paradigm (ad libitum for 1 week vs once a day for 2 days i.p. injection) and/or other experimental conditions (e.g., female C57BL/6 J mice at P60-90^52^ vs male and female CD-1 mice at P40 in our study).

Our acute PAE model mimics short-term binge drinking during the second trimester. While it does not mimic the most common drinking pattern of pregnant women—drinking early in pregnancy before the pregnancy is known and then stopping alcohol consumption—it represents a real-world scenario, where a subset of women consume alcohol in unusual patterns—for example, abstaining in the first trimester then drinking at high levels in the second or third trimester.^53^^,^^54^ Though small in proportion, the total number of such women should be enormous. Binge-drinking models enable the study on acute, high-dose effects that drive key mechanisms of cognitive impairments in FASD, and second-trimester exposure models target a critical window when the brain undergoes multiple developmental events that are vulnerable to alcohol, making it suitable for studying the mechanisms of abnormal brain development caused by PAE.^55^ Intraperitoneal injection models offer precise control over the timing, dosage, and duration of alcohol exposure, which minimizes variability and confounding factors, providing clear insights into causal mechanisms. These models share high consistency with reports on human cohorts that include a variety of drinking patterns.^56^^,^^57^ Thus, beyond some limitations, this model has many advantages for researchers studying the mechanisms of PAE-induced brain abnormality. Nevertheless, future comparisons with different models, such as chronic-drinking models and free-drinking models, will be essential to examine the applicability of our findings to a wider range of FASD patients.

Our water T-maze test revealed no discernible differences between control and PAE mice in the spatial learning phase (Figs. 1, 5). The effects of PAE on spatial learning vary across the literature and are potentially influenced by factors such as the timing of alcohol exposure, dosage, type of behavior test, and age during behavior tests.^58^ In contrast, reversal learning was found to be affected by PAE (Figs. 1, 5), consistent with previous PAE studies.^2^^,^^59^ Previously, we have shown that PAE differentially affects the expression of KCNN2 across brain regions; the expression level remained normal in the hippocampus while increased in the motor cortex,^17^ possibly due to regional differences in the developing timing relative to the timing of exposure, endogenous KCNN2 regulatory mechanism, cell subtypes, neurovascular coupling, etc. In addition, the elevated KCNN2 levels in pyramidal neurons in the motor cortex were found to increase the amplitude of medium afterhyperpolarization and alter firing patterns in those neurons as a potential contributors to motor learning deficits in PAE mice.^17^ In the present study, we noted an increase in KCNN2^+^ cells in layer V of the cingulate and prelimbic regions of the PFC, with a modest rise in the orbitofrontal and infralimbic regions in PAE mice (Figure 2). Since reversal learning requires cohesive network activity among the PFC, striatum, and amygdala,^60^ these results suggest that elevated KCNN2 levels in the PFC may contribute to reversal learning deficits by increasing the amplitude of medium afterhyperpolarization, thereby altering the firing patterns of pyramidal neurons. This model aligns with our finding that blocking KCNN2 with Lei-Dab7 mitigates the cognitive inflexibility in PAE mice (Figure 5). Similar findings have been reported in a 2-hit mouse model of maternal immune activation and juvenile social isolation that mimic the symptoms of schizophrenia, where enhanced medium afterhyperpolarization in neurons in layer V of the PFC due to increased KCNN3 (SK3) channel expression has been suggested to contribute to their cognitive inflexibility.^61^ However, to formally validate our model, further experiments such as region-specific manipulation of KCNN2 would be imperative.

KCNN channels form both homo- and heterotetramers to create functional channels.^62^^,^^63^ The unique expression patterns of each of the 4 members of KCNN channels contribute to specific brain functions,^64^ and genetic variants and alterations in their expression levels (both increases and decreases) have been implicated in various neurological and psychiatric diseases.^65^ In this study, varying degrees of KCNN2 upregulation specific to each subregion of the PFC were observed in PAE animals (Figure 2). Basal KCNN2 expression levels also differed among these subregions in normal animals (Figure 2). Different subregions of the PFC in rodents are associated with distinct processes of behavioral flexibility. For instance, the orbitofrontal region plays a role in reversal learning of a previously acquired rule,^66^ while the prelimbic and infralimbic areas are essential for strategy switching (or rule shifting)^67^^,^^68^ but not for reversal learning.^67^ The heterogeneity in KCNN2 expression changes by PAE across PFC subregions, in conjunction with their distinct roles in behavioral flexibility, therefore suggests a nuanced interplay between KCNN2 expression and various cognitive processes.

Our results showed that intranasally administered Lei-Dab7 effectively reaches its target regions, including the PFC (Figs. 3, 4) in addition to the motor cortex, where KCNN2 is upregulated.^17^ This result encourages us to profile pharmacokinetics in the brain, cerebrospinal fluid, and blood. The potential of intranasal delivery in treating a broad spectrum of neurological disorders, especially those related to memory and cognition, is noteworthy.^28^ Historically, intranasal administration has gained attention in clinical trials as an effective delivery method, especially for small molecules and peptides.^69^ For instance, a synthetic leptin-like peptide has been shown to be 4 times more bioavailable to the brain when delivered intranasally than through other methods such as subcutaneous or intramuscular administration.^70^ There is also compelling evidence of intranasal insulin improving memory in older adults affected by Alzheimer disease or mild cognitive impairment.^31^ Nasal application of oxytocin as a treatment of neurodevelopmental disorders such as autism spectrum disorder has also been explored.^71–75^ While its single administration has proven promising, the efficacy of repeated doses remains inconsistent.^30^^,^^33^^,^^71–75^ Similarly, our study, which focused on the effects over a short duration (Figure 5), underscores the importance of evaluating the optimal dosage and duration of Lei-Dab7 treatment. Enhancing the delivery efficiency would involve optimizing the delivery vehicle. The incorporation of absorption enhancers, such as torsemide, bile acids, and various phospholipids, may improve drug bioavailability.^76^^,^^77^ While the stability of Lei-Dab7 with its 3 disulfide bonds is commendable, it could be further refined by adjusting properties such as osmolarity, pH, and viscosity. Additionally, recent studies indicate that nasal administration in powder form may offer better outcomes than soluble forms.^78^

Taken together, our findings advocate for the therapeutic potential of Lei-Dab7 or other KCNN2 blockers for FASD and possibly other neurodevelopmental diseases that similarly involve KCNN2 upregulation, such as Angelman syndrome.^21^ Lei-Dab7 stands out for its high potency and specificity in blocking the KCNN2 channel. This is especially notable compared with other blockers such as apamin, scyllatoxin, bicuculline, UCL 1684, and UCL 1848, which often serve as nonspecific KCNN (SK) channel blockers.^79^ Despite outstanding properties, Lei-Dab7’s bioavailability, efficacy, and toxicity as a therapeutic agent have remained undetermined. Our study provides the first clues about these. Demonstrated benefits in both in vivo and in vitro settings without apparent toxicity are encouraging. Tremor has been reported in *Kcnn2*-deficient (frissonnant) mice,^80^ but no such abnormalities have been reported in *Kcnn2* knockout (null) mice.^81^ In rat studies, no negative effects of Lei-Dab7 on learning have been found^82^; and apamin, a less selective KCNN2 blocker, has rather been shown to promote learning.^26^^,^^82^^,^^83^ Thus, as we also observed no apparent adverse effects in control animals under our experimental condition (Figs. 5, S4), KCNN2 blockade by Lei-Dab7 may have very low toxicity at its effective dose. As we move forward, rigorous dose-escalating tests will be indispensable. Ziconotide, a intrathecal analgesic, has proven that venom-derived peptide same as Lei-Dab7 can safely be used as a drug.^84^ It is also worth noting that bee venom, which acts as a pan-SK channel blocker, has been safely used in adult clinical trials for Parkinson disease.^85^ Nevertheless, given the associations of KCNN2 channel activity with conditions like atrial fibrillation and epilepsy,^65^^,^^86–88^ close monitoring of Lei-Dab7’s effects, especially on cardiac functions, will be imperative. Our study explores the therapeutic potential of postnatal KCNN2 blockade for cognitive deficits in FASD. However, since Kcnn2 mRNA expression begins in the embryonic period,^89^^,^^90^ PAE may disrupt its expression and function very early and affect brain development. Given the potential for bioelectrical modulation targeting ion channels to prevent alcohol-induced abnormalities in early brain development,^91–93^ further research on this aspect of KCNN2 channel modulation would also be warranted.

## Supplementary Material

Supplementary_Information_pyaf055

## Data Availability

The data underlying this article are available upon request to the corresponding author.
