# Supplementary material for: Intranasal Administration of KCNN2 Blocking Peptide Improves Deficits in Cognitive Flexibility in Mouse Model of Fetal Alcohol Spectrum Disorders
Source: Int J Neuropsychopharmacol. 2025 Aug 8;28(9):pyaf055. doi: 10.1093/ijnp/pyaf055 (PMC12418948; doi:10.1093/ijnp/pyaf055)

**Supplementary Figure 1. Prenatal ethanol exposure does not cause depression-like behavior assessed by the forced swim test. (A, B)** Experimental timeline (A) and categories of behaviors observed in the forced swim test (B). **(C, D)** The durations of swimming (C) and immobility (floating) (D) are unaffected in PAE mice compared to controls. P = 0.93 (C) and P = 0.87 (D) by two-tailed Student’s *t*-test (n = 10 animals from 5 litters per group [including both sexes]). Data are presented as the mean with standard error.


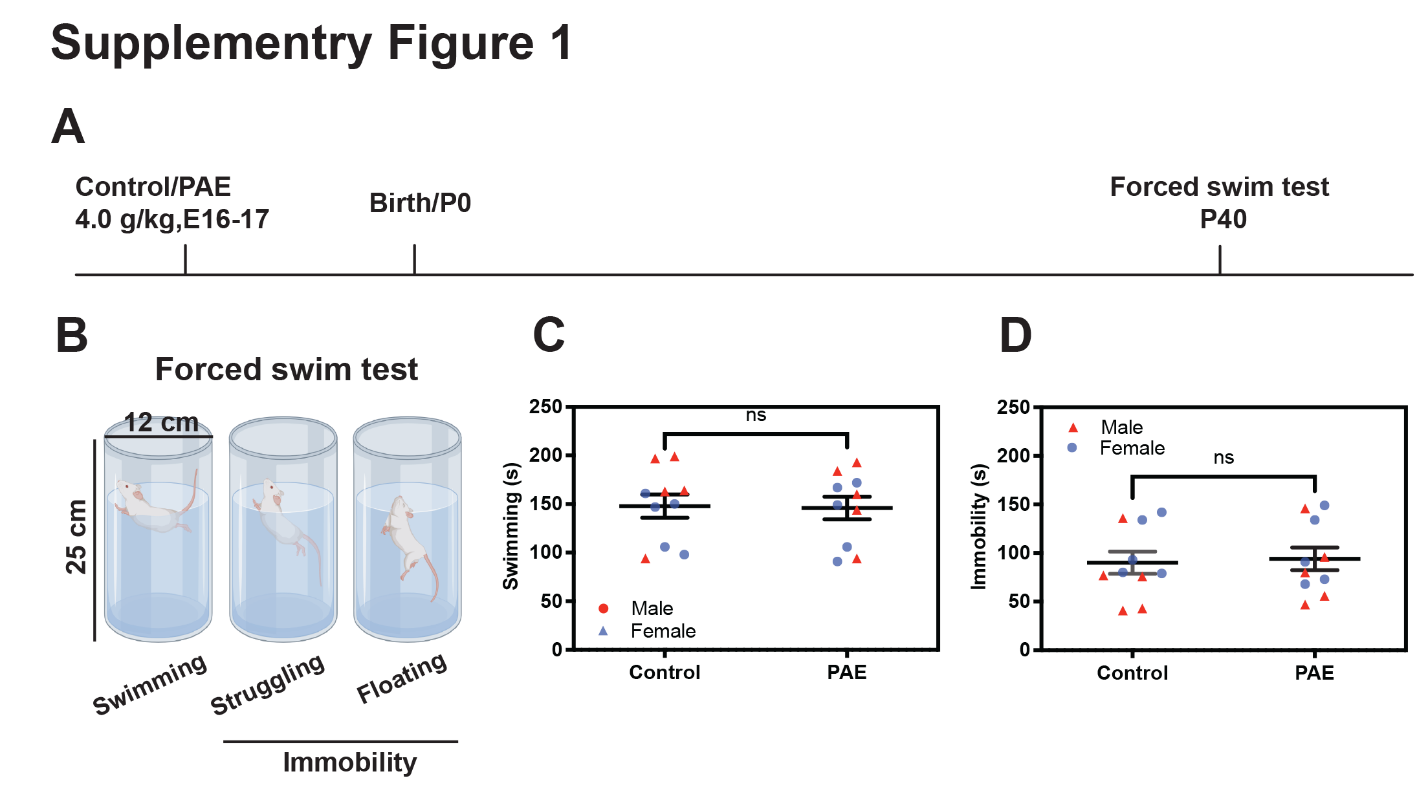


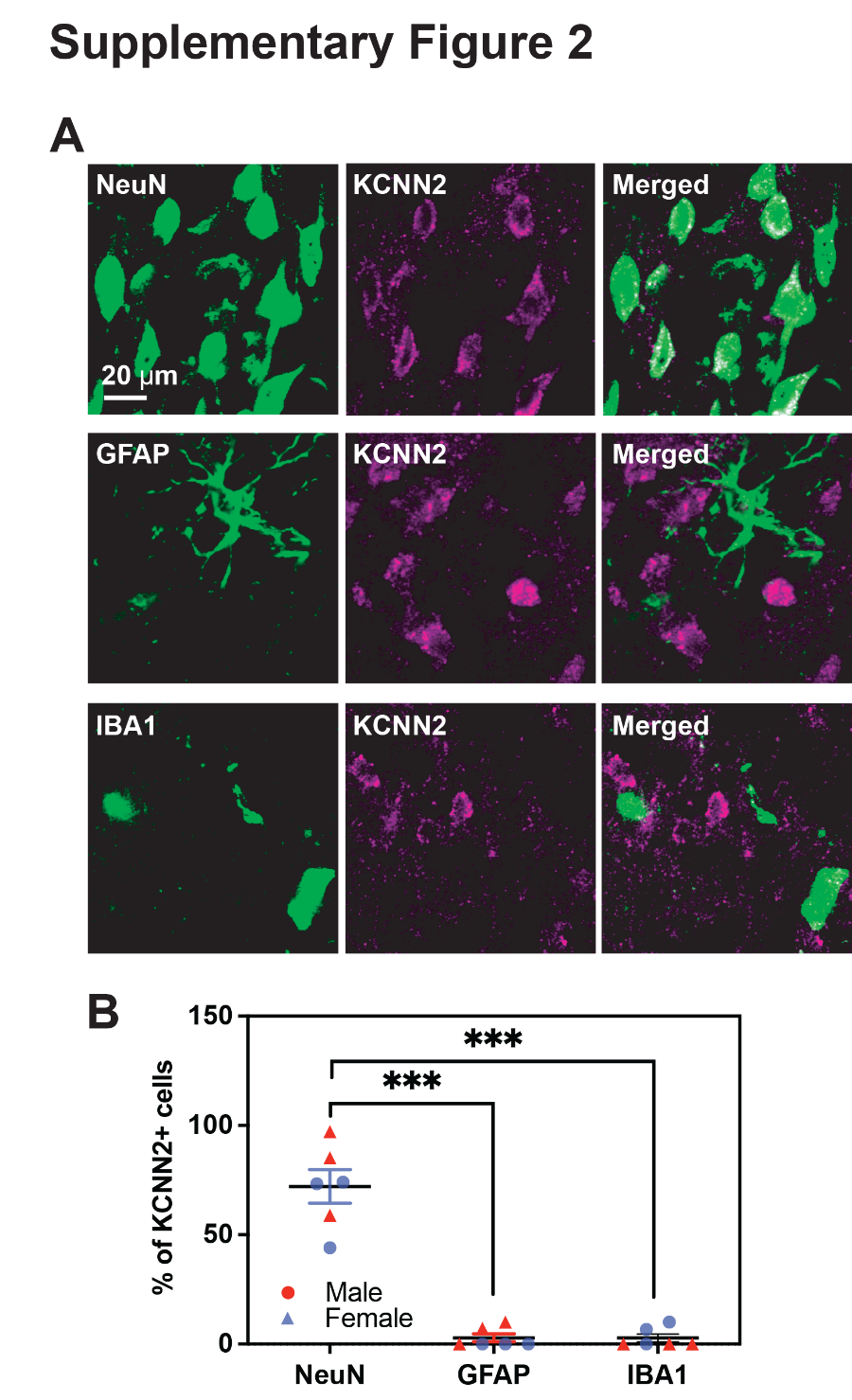
**Supplementary Figure 2. Neurons express KCNN2 in the PFC of PAE mice. (A)** Immunohistochemistry reveals that KCNN2 is expressed in most NeuN^+^ neurons, but not in GFAP^+^ astrocytes or IBA1^+^ microglia in layer V of the PFC in PAE mice at P40. **(B)** Percentages of KCNN2^+^ cells in cells expressing the indicated cell type markers in layer V in PAE mice. F(2,15) = 73.06, ***P < 0.001 by one-way ANOVA (n = 6 animals from 3 litters per group [including both sexes]). Data are presented as the mean with standard error.

**Supplementary Figure 3. Concentration-response relationships of Lei-Dab7 and Tamapin on the Inhibition of KCNN2 channels.** **(A, B)** The concentration-response relationships of Lei-Dab7 (A) and Tamapin (B) on the inhibition of KCNN2 (SK2) channels examined by the thallium flux assay are presented. Data are presented as the mean with standard error (n = 4 replicates).


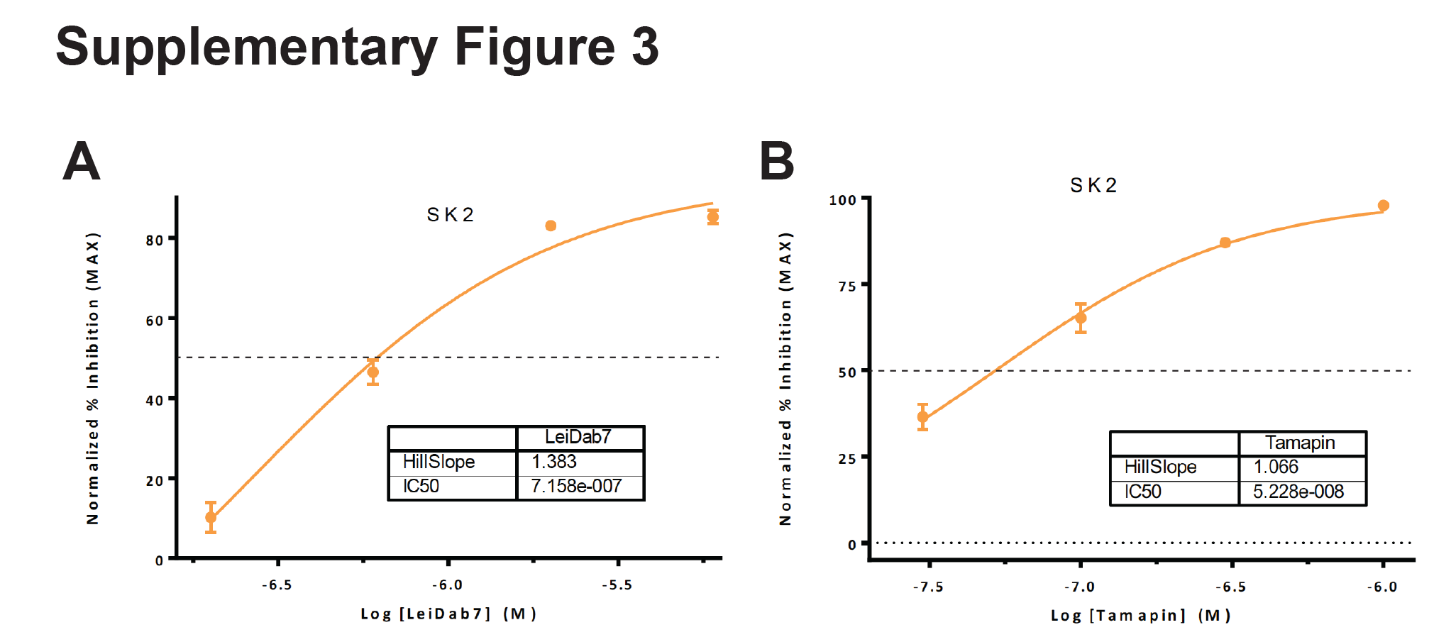


**Supplementary Figure 4. Lei-Dab7 does not affect animal’s physiological status at P40.** Pulse oximetry, heart rate, and body temperature were recorded at 30 minutes and 24 hours post administration of vehicle control (30 minutes only) or Lei-Dab7 (30 minutes, 24 hours). Siblings from the same mother were used as the control for the Lei-Dab7 treatment. There were no statistically significant differences between the indicated groups as determined by One-way ANOVA (n = 6 animals from 3 litters per group [including both sexes]). Data are presented in box plots. The line within the box indicates the median, and the upper and lower edges of the box represent the 25th and 75th percentiles, respectively. The upper and lower whisker boundaries indicate the 10th and 90th percentiles, respectively, and dots indicate outliers.


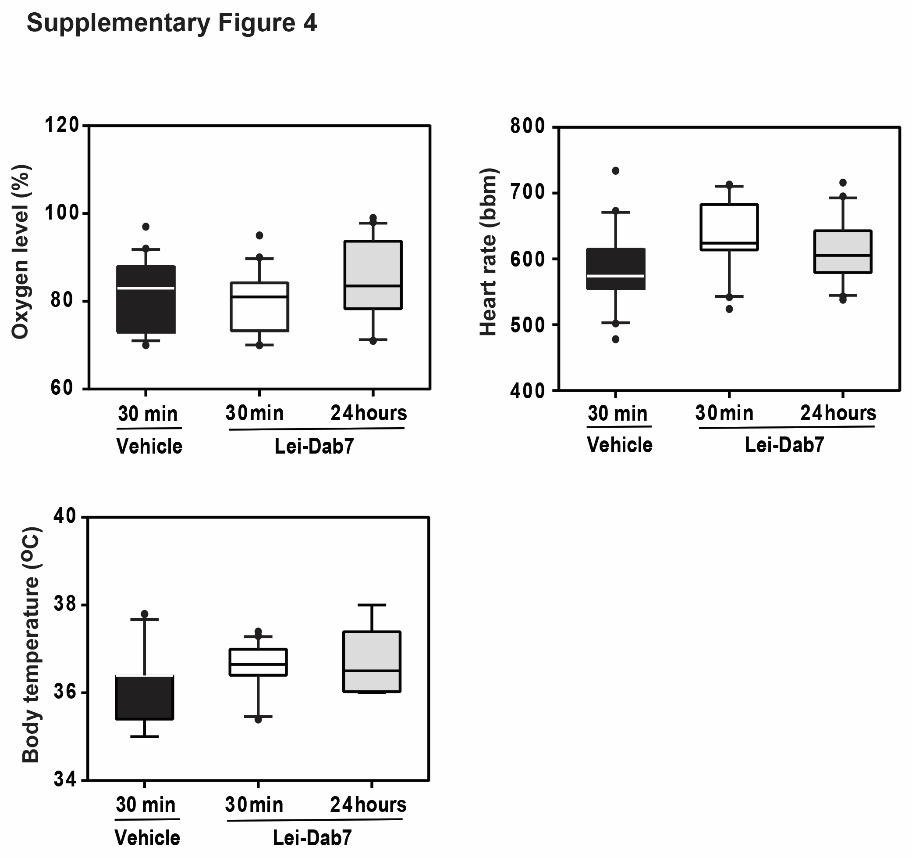

Supplement: Supplementary_Information_pyaf055 [file supplementary_information_pyaf055.docx]
